# Metals and Neurodegeneration

**DOI:** 10.12688/f1000research.7431.1

**Published:** 2016-03-17

**Authors:** Pan Chen, Mahfuzur Rahman Miah, Michael Aschner

**Affiliations:** 1 Department of Molecular Pharmacology, Albert Einstein College of Medicine, New York, New York, USA

**Keywords:** metal accumulation, neurological disorders, Alzheimer’s disease, neurodegeneration, Huntington’s disease, Parkinson’s disease

## Abstract

Metals play important roles in the human body, maintaining cell structure and regulating gene expression, neurotransmission, and antioxidant response, to name a few. However, excessive metal accumulation in the nervous system may be toxic, inducing oxidative stress, disrupting mitochondrial function, and impairing the activity of numerous enzymes. Damage caused by metal accumulation may result in permanent injuries, including severe neurological disorders. Epidemiological and clinical studies have shown a strong correlation between aberrant metal exposure and a number of neurological diseases, including Alzheimer’s disease, amyotrophic lateral sclerosis, autism spectrum disorders, Guillain–Barré disease, Gulf War syndrome, Huntington’s disease, multiple sclerosis, Parkinson’s disease, and Wilson’s disease. Here, we briefly survey the literature relating to the role of metals in neurodegeneration.

## Introduction

Metals are a component of the earth’s crust and exist in the water, air, and a variety of ecosystems. They can generally be divided into two groups: essential and non-essential metals. Essential metals include chromium, cobalt, copper (Cu), iron (Fe), lithium, magnesium, manganese (Mn), nickel, selenium, and zinc (Zn). These trace metals usually act as a cofactor of enzymes to regulate cellular activities. For example, Mn is required for the activity of arginase, hydrolases, lyases, glutamine synthetase, and superoxide dismutase (SOD) ^1^. Thus, these metals are involved in a whole host of physiological processes, such as electron transport, oxygen transportation, protein modification, neurotransmitter synthesis ^2, 3^, redox reactions, immune responses, cell adhesion, and protein and carbohydrate metabolism ^1^, to name a few. Metals accumulate in the brain, indicating their important roles in the nervous system. Deficiency of these metals has been associated with various neurological diseases. For example, Fe deficiency is related to restless leg syndrome, pediatric stroke, breath-holding spells, pseudotumor cerebri, and cranial nerve palsy ^4, 5^.

Although metals are important for animals and plants, they usually are required in trace amounts. Excessive metal levels accumulate in various organs, including the brain. Elevated levels of metals may induce various detrimental intracellular events, including oxidative stress, mitochondrial dysfunction, DNA fragmentation, protein misfolding, endoplasmic reticulum (ER) stress, autophagy dysregulation, and activation of apoptosis ^6– 13^. These effects may alter neurotransmission and lead to neurodegeneration, which can manifest as cognitive problems, movement disorders, and learning and memory dysfunction. To date, metal-induced neurotoxicity has been associated with multiple neurological diseases in humans, including Alzheimer’s disease (AD), amyotrophic lateral sclerosis (ALS), autism spectrum disorders (ASDs), Guillain–Barré disease (GBD), Gulf War syndrome (GWS), Huntington’s disease (HD), manganism, multiple sclerosis, Parkinson’s disease (PD), and Wilson’s disease (WD) ^3, 14– 18^. Here, we address the neurotoxicity of several metals as well as the human neurological diseases associated with these metals.

## Essential metals

### Copper

Cu is an essential trace element and a transition metal required for physiological activities in mammals. Cu acts as a cofactor of various enzymes (such as cytochrome *c* oxidase and SODs), playing an important role in electron transport, oxygen transportation, protein modification, and neurotransmitter synthesis ^2, 3^. However, elevated Cu levels may result in the generation of reactive oxygen species (ROS), DNA damage, and mitochondrial dysfunction ^3^. Excessive Cu has been associated with AD, ALS, HD, PD, WD, and prion diseases in humans ^3, 16, 19^. Cu may enhance the self-aggregation of amyloid precursor proteins and β-amyloid peptide ^20^, and increased levels of Cu in cerebrospinal fluid have been found in some patients with AD ^21^. Similarly, Cu interacts with α-synuclein and promotes its aggregation, which could result in PD ^22^. Gain-of-function mutations in Cu/Zn-SOD might result in oxidative stress through production of free radicals, possibly leading to motor neuron degeneration in patients with ALS ^3, 21^. It is also noteworthy that metals such as Cu and Zn are controlled by overlapping proteins, such as metallothionein ^23^. Notably, Cu levels are also higher in HD patients compared with controls ^24^.

Prion diseases are characterized by altered structure, changing from a normal cellular isoform (PrP ^C^) to an abnormal scrapie isoform (PrP ^Sc^), which contains high-affinity Cu-binding sites. The binding of Cu enhances the stability of prion proteins, making them resistant to proteasomal degradation and leading to neurodegeneration ^3^. However, the binding capability of Cu to the prions does not explain its role in the progression of prion disease. It is noteworthy that metal mixtures, such as silver and Cu, have been shown to change the affinity of PrP ^C^ to Cu ^19^. In addition, an inverse correlation between Cu and Mn has been noted in patients with prion disease ^25, 26^. Cu does not appear in the formation of PrP ^Sc^, and increases in Cu in the diet have been associated with delays in the onset of prion disease ^27– 29^. Given both the beneficial and neurotoxic effect of Cu in the brain, new biomarkers and improved measurement of Cu trafficking are needed to predict potential risks, and novel therapeutics need to be developed for Cu-induced neurotoxicity and cognitive dysfunction.

### Iron

Fe is an essential metal, serving as a cofactor for a variety of enzymes and proteins, most prominently hemoglobin ^30^. Fe’s ability to interact with oxygen makes it important for oxygen transport in the cellular respiration pathway as well as for a variety of redox reactions ^31^. Exposure to Fe is primarily through food consumption, although toxic levels of Fe accumulation are usually due to disrupted Fe homeostasis and metabolism in the brain ^32, 33^. Hemolysis, the breakdown of red blood cells, in the young brain with an immature blood-brain barrier (BBB) can also lead to aberrant Fe accumulation, which results in neuronal damages ^34^. Fe accumulation can cause increased ROS levels, lipid peroxidation, protein oxidation, DNA damage, dopamine autoxidation, and mitochondrial fragmentation ^35– 38^.

Fe dyshomeostasis has been linked to a variety of neurological disorders, including PD, AD, HD, ALS, and neurodegeneration with brain iron accumulation (NBIA) ^36, 39– 42^. Increased brain Fe deposition has been observed and Fe has been shown to promote aggregation of α-synuclein, which is found in patients with PD ^43– 45^. Increased Fe accumulation in the brains of patients with AD has also been observed along with evidence of Fe contributing to β-amyloid aberrant aggregation and toxicity, a hallmark of the disease ^46– 48^. Increased Fe is also seen in patients with HD, and Huntingtin (htt), the central protein in HD pathology, is thought to mediate Fe homeostasis ^49, 50^. Patients with ALS show accumulation of Fe in the motor cortex, and *SOD-1*, a gene mutated in patients with ALS, has been shown to cause damage via Fe-induced oxidative stress ^51– 54^. Finally, NBIA, as the name implies, is an umbrella of heterogeneous neurodegenerative diseases that present with simultaneous Fe accumulation. Genetic studies indicate that the majority of the genes mutated in patients with NBIA are related not to Fe homeostasis but rather to autophagy, mitochondria metabolism, and lipid metabolism. This suggests that Fe accumulation in patients with NBIA may not be the initial insult of a disease pathology but rather a downstream phenomenon that serves to exacerbate already-present issues ^41^. Alternatively, these genes may have currently unknown Fe regulatory functions that have yet to be examined. This question of whether Fe is part of the cause of a disease, or vice versa, continues to be a point of controversy.

### Manganese

Mn is a trace element and nutrient necessary for biological processes within the human body ^55^. Low concentrations of Mn are essential to the body; Mn serves as a cofactor in a variety of metalloproteins, including MnSOD and arginase, and is important in the function of a variety of enzymes, including glutamine synthetase, hydrolases, and lyases ^55, 56^. Exposure to high levels of Mn may be toxic. Consumption of food is the primary route for entrance into the body ^56, 57^. Mn can be inhaled as well, and this serves as an occupational hazard for those who work in welding and mining industries ^1, 58, 59^. Soy-based infant formula has been suggested as an exposure route for excess Mn, which may lead to mild neurological defects during critical stages of development ^60, 61^. Chronic Mn exposure can cause debilitating neurological effects. Mn overexposure leads to a type of parkinsonism known as manganism and has been suggested as part of PD etiology as well ^55, 59^. Manganism is characterized by tremors, lethargy, and speech impediments, with the occasional accompaniment of psychosis ^60, 62^. Mn is elevated in dopaminergic (DAergic) neurons of the substantia nigra, providing a possible basis for the motor deficits observed in manganism ^63^. Elevated Mn has been shown to affect a variety of cellular processes, including increased levels of transcription for ER-related genes ^64^, ROS production ^65^, mitochondrial dysfunction ^66^, autophagy ^67^, altered acetylcholinesterase (AChE) activity ^68^, changes in cyclic AMP (cAMP) signaling ^69^, iron dyshomeostatis ^70^, and dysfunctional astrocytic activity ^61^. Many markers of programmed cell death, including increased TUNEL (terminal deoxynucleotidyl transferase dUTP nick end labeling) staining, internucleosomal DNA cleavage, activation of JNK, p38, the apoptotic initiator caspase-12, and pro-apoptotic effector caspase-3, have been observed in neurons in the presence of Mn exposure ^9, 71^. Specifically, Mn has been shown to induce proteolytic cleavage of protein kinase C-δ (PKC-δ) as an essential player in Mn-induced neuronal death and in the neuroprotective activity of α-synuclein protein aggregation upon chronic Mn exposure ^72– 74^. The induction of apoptosis is possibly mediated through ER stress and autophagy. In DAergic SH-SY5Y cells exposed to Mn, the levels of ER stress response proteins, including the ER chaperone GRP94 and the pro-apoptotic GADD153/CHOP protein, as well as phosphorylated eIF2α (eukaryotic translation initiation factor 2α) are increased significantly ^9^. In Mn-treated DAergic neurons, increased abnormal lysosomes, decreased expression of autophagy-related protein Beclin1, and activation of mammalian target of rapamycin (mTOR)/p70 ribosomal protein S6 kinase (p70s6k) signaling have been observed, possibly leading to DAergic neurodegeneration ^8^.

In addition, PD-related mutant genes (such as ATPase *12A3*) have been shown to alter Mn homeostasis, supporting the link between Mn dyshomeostasis and PD. Interestingly, the newly identified Mn transporter SLC30A10 has been associated with dystonia, parkinsonism, and hypermanganesemia when mutated ^75– 77^. This protein is expressed at high levels in the liver and the basal ganglia ^77^. Loss-of-function mutants prevent Mn excretion and result in high levels of plasma Mn, which eventually accumulates in the basal ganglia of the brain ^75, 77^. Indeed, Mn can also catalyze the autoxidation of dopamine, whose toxic metabolites can wreak havoc on DAergic cells, suggestive of similar pathology between PD and manganism ^37, 59^. In regard to prion disease, a protective role for prion protein against Mn neurotoxicity has been suggested upon short-term exposure to Mn, although prolonged Mn exposure was shown to promote the stabilization and aggregation of the infectious protein ^78, 79^. Although Mn is an essential metal, Mn homeostatic and signaling pathways are still being delineated, and further investigation may give insight into not only Mn regulation but also manganism and other forms of parkinsonism.

### Zinc

Zn is an essential trace metal (the second most abundant transition metal after Fe) required for humans and many other living organisms. It is a cofactor for over 300 enzymes and metalloproteins ^80^, regulating gene transcription and the antioxidant response. The majority of Zn is in the testes, muscle, liver, and brain ^81^. In childhood, Zn deficiency affects mental and physical development as well as learning abilities ^81, 82^. However, excess levels of Zn suppresses Cu and Fe absorption ^83^, promoting ROS production in the mitochondria, disrupting activities of metabolic enzymes, and activating apoptotic processes ^6^. Disruption of Zn homeostasis has been associated with AD, brain trauma, cerebral ischemia, epilepsy, and vascular-type dementia (VD) ^6, 81^. At low concentrations (a few micromolar), Zn suppresses β-amyloid-induced neurotoxicity by selectively precipitating aggregation intermediates ^84, 85^. However, at high levels, the binding of Zn to β-amyloid may enhance formation of fibrillar β-amyloid aggregation, leading to neurodegeneration ^86, 87^. Zn also plays a critical role in ischemia-induced neuronal death and the pathogenesis of VD, evidenced by a correlation of increased Zn concentration with glutamate being packaged into synaptic clefts during membrane depolarization under ischemic conditions ^81, 88^. Future research should assess the intracellular activities of supplemental and neurotoxic Zn in the nervous system and investigate optimal Zn doses needed for different groups of people, especially infants and children.

## Non-essential metals

### Aluminum

Aluminum (Al) is the third most abundant element and the most abundant metal in the earth’s crust. It has a wide range of uses, including in food preservation, cans, cookware, cars, and vaccine adjuvants, to name a few ^14^. The exact function of Al in animals remains unknown. Al is highly reactive with carbon and oxygen, making it toxic to living organisms. In humans, Al from dietary intake and environmental exposure is rapidly cleared by the kidney. However, Al salts in vaccine adjuvants remain biologically available and accumulate in the nervous system. Al has been associated with AD, ALS, ASDs, GBD, multiple sclerosis, and GWS in humans ^14, 15^. In patients with dialysis-associated encephalopathy, Al accumulation was found in the brain together with β-amyloid peptide accumulation ^89, 90^. Interestingly, the symptoms quickly abated after removal of Al from the dialysis solution ^91^. Recently, a meta-analysis showed that chronic Al exposure increased the risk of AD by approximately 70% ^92^. In addition, a correlation between the number of children with ASD and exposure to Al-adjuvanted vaccines was observed ^14^; higher levels of Al were found in the hair, blood, and urine of children with autism ^93^. In mice, injection of Al hydroxide results in loss of long-term memory, increased anxiety, and neuronal death in the spinal cord and motor cortex ^14^. The neurological damage may be due to oxidative stress ^12, 13^ and mitochondrial dysfunction ^94– 96^. However, the results of many of these reports do not address confounding variables such as genetic backgrounds that may predispose an organism to the susceptibility of Al-induced neurological damage. Taken together, genetic polymorphisms combined with environmental factors likely trigger Al-induced neurotoxicity. Future studies could be profitably directed at investigating the relationship between Al exposure and genetic risk factors of these neurological diseases.

### Arsenic

Commonly used as a wood preservative in the past and known for its contamination of groundwater, Arsenic (As) is a toxic metalloid and well-known carcinogen, which over 200 million people worldwide are chronically exposed to ^97– 100^. Research suggests that As exposure induces mitochondrial oxidative stress, imbalance of intracellular Ca ^2+^, disruption in ATP production, altered membrane potential, changes in cytoskeletal morphology, and neuronal cell death, among other effects ^97, 101^. Studies have shown that early-life exposure to As can cause lower brain weight and a reduction in glia and neurons ^98^. As exposure is associated with AD and ALS ^101, 102^. Dimethylarsenic acid, a metabolite of As in humans, has been shown to increase β-amyloid levels, a key feature of AD ^103^. Translocation of neurofilament (NF) was inhibited by As exposure and is linked to decreased NF content at peripheral nerves. This may be important in understanding the aberrant NF distribution that is a hallmark of ALS ^104^. Owing to the persistent exposure of As to the human population, further investigation into the mechanisms of As poisoning is warranted.

### Cadmium

Cadmium (Cd) is a non-essential transition heavy metal and known human carcinogen ^105^. It can enter the peripheral and central neurons from the nasal mucosa or the olfactory bulb, which damages permeability of the BBB ^106^. Miners, welders, smokers, and workers in battery production are at risk of high Cd exposure ^106^. In cells, Cd can induce oxidative stress, suppress gene expression, and inhibit DNA damage repair and apoptosis ^107^. Chronic exposure to Cd may severely interfere with normal function of the nervous system, and infants and children are more susceptible than adults ^106^. Cd is a possible etiological factor of neurodegenerative diseases, including AD ^108^ and PD ^17^. The symptoms include headache, megrim, olfactory dysfunction, slowing of vasomotor functioning, decreased equilibrium and learning ability, and PD-like symptoms ^106^. Jiang *et al.* found that Cd accelerates self-aggregation of Alzheimer’s tau peptide R3 ^108^, and it has been reported in a case study that a 64-year-old man developed PD symptoms 3 months after acute exposure to Cd fumes ^17^. In zebrafish, Cd exposure resulted in decreased head size and unclear brain subdivision boundaries in the mid-hindbrain region ^109^. In addition, Cd exposure has been shown to result in morphological changes of rat cortical neurons (axons and dendrites) ^110^ and inhibited neurite outgrowth in PC12 cells ^111^. Owing to Cd’s role as a carcinogen, the neurotoxicity induced by Cd is underestimated. Thus, future research should further investigate the mechanism of neurotoxic effect of Cd, especially that of chronic low-level Cd exposure.

### Lead

Lead (Pb) is a non-essential heavy metal and a ubiquitously present pollutant in the ecosystem. In humans, inhalation and oral ingestion are the major routes of Pb exposure. In the body, Pb can be excreted in urine and bile, and some Pb can bind to red blood cells and eventually accumulate in bone ^112^. Pb exposure results in oxidative stress, mitochondrial dysfunction, changes to the Golgi apparatus, and increased gliofilaments in astrocytes ^113^. It also disrupts Ca ^2+^ homeostasis ^114^, interferes with the phosphorylation of PKC ^115^, and decreases nitric oxide production ^116^. The main target of Pb-induced toxicity is the nervous system, and children are particularly sensitive to Pb intoxication. The hippocampus is the primary region of Pb accumulation, although the metal may also accumulate in several other brain regions ^117^. It has been reported that Pb exposure results in deficits in intelligence, memory, executive functioning and attention, processing speed, language, emotion, and visuospatial and motor skills ^112^. For example, in children, Pb exposure results in decreased intellectual ability in a dose-dependent manner ^118, 119^, impaired verbal concept formation ^120^, grammatical reasoning difficulty ^121^, poor command following ^122^, and so on. In adults, workers with occupational exposure to Pb have shown impairment of verbal memory and visual memory performance ^123^, lower decision-making speed ^118^, deficit in visuomotor coordination ^120^, and increased interpersonal conflict ^124^. Given the severe health issues caused by Pb, novel techniques for early diagnosis of Pb exposure (especially during pregnancy and childhood) and effective treatment after Pb exposure should be the focus of future research on Pb.

### Methylmercury

Methylmercury (MeHg) is a xenobiotic toxic organic metal compound derived from inorganic mercury (Hg). Hg finds itself in our environment primarily through anthropogenic sources such as industrial waste, coal mining, and natural sources such as volcanoes and forest fires that release Hg back into the atmosphere ^125^. Hg that pollutes the aquatic arena through these paths can be readily methylated into MeHg by sulfate-reducing bacteria and a variety of other anaerobic bacteria ^126^. MeHg bioaccumulates in the aquatic food chain, and seafood consumption remains one of the main forms of exposure for humans ^127^. MeHg has a high affinity for sulfur and can cross the BBB by binding onto thiol groups of proteins; it can also bind to lone cysteine, mimicking the structure of methionine, allowing for the possibility of uptake by amino acid transporters ^128, 129^. MeHg can accumulate in the brain and has led to epidemics in the past at Minamata Bay and Iraq, where those affected, particularly children, presented with a variety of central nervous system (CNS) disorders, including ataxia, paralysis, retardation, dysarthria, dysesthesia, and cerebral palsy ^130^. A case study of autopsies in a family that was exposed to high levels of MeHg revealed that exposure to MeHg leads to inorganic Hg buildup in the brain. Furthermore, cerebellar and occipital lobe atrophy was observed in patients who had experienced motor and vision issues. Differential distribution of Hg content was also observed, and the greatest amounts were in the occipital lobe and cerebellum and basal ganglia, reflective of the areas important for vision and movement ^131^. At the cellular level, MeHg is known to affect a variety of neuronal activities including dopamine metabolism ^132^, neural stem cell differentiation ^133^, generation of ROS ^134^, increased calcium influx ^135^, aberrant autophagy ^136, 137^, DNA damage, and mitochondrial dysfunction ^138^. In addition, MeHg exposure has been shown to increase β-amyloid in the hippocampus and decrease it in the cerebrospinal fluid, both hallmarks of AD ^139^. Currently, treatment options for those exposed to MeHg are not prevalent. Prevention of exposure has been the major development in the past few decades whereby government advisories have been established to inform and protect their respective populations ^140^. Limitations on fully understanding the effect of MeHg on the CNS include the confounding beneficial nature of seafood by which MeHg usually enters the human body ^141^.

### Thallium

Thallium (Tl), a naturally occurring trace element, is an extremely toxic heavy metal, sparsely found in the earth’s crust ^142, 143^. Sources of Tl include industrial processing of cement and non-industrial means such as rodenticide and eating foods from contaminated soils. Tl can make its way into the body not only through consumption but also via the skin and inhalation ^142, 144^. Non-neurological symptoms include alopecia, hepatic dysfunction, gastroenteritis, and Mees’ lines, and CNS-related disorders include polyneuropathy, cranial nerve deficits, paresthesia, loss of sensation, ataxia, and psychosis ^142, 144– 146^. Victims of Tl poisoning complain of peripheral neuropathy ^146, 147^. It has been shown that Tl exposure can lead to inhibition of AChE, an enzyme catalyzing breakdown of the neurotransmitter acetylcholine (Ach), which may explain peripheral neuropathy ^148^. Studies have shown that Tl concentrations in the brain are lower than in other parts of the body ^149, 150^ and that the highest concentrations in the brain are in the hypothalamus ^150^. Tl ^+^ interferes with a host of K ^+^-dependent processes because of similarities in size and the univalent nature of the two ions; one of the affected processes is ATP generation ^142, 149, 151^. At the cellular level, higher levels of Tl have been shown to cause a decrease in ATP production, increase in ROS formation, glutathione oxidation, and decreases in dopamine and serotonin levels in the brain ^142, 151, 152^. Owing to available treatments for this xenobiotic metal and lack of major risk of exposure to the general public, clarity in signaling pathways remains an issue. Furthermore, the complicated symptoms may cause misdiagnosis of Tl poisoning, which points to the need for health professionals to be more aware of the features of Tl-induced poisoning and neurotoxicity ^153^.

### Neurotoxicity induced by metal mixture

The neurotoxicity of metals is usually studied on an individual metal basis. However, the fact is that we are exposed to an environment of mixed metals, which makes it more complicated to study. It is noteworthy that changes in the level of one metal may have significant effect on the homeostasis of other metals, given that various metals (such as Mn, Fe, Cd, Cu, and Zn) are transported by shared transporters or controlled by overlapping signaling pathways. There are limited studies focusing on the combined effect of mixed metal exposure. The neurotoxicity of Pb with other metals (As, Cd, Hg, and Mn) is well studied ^154^. Prenatal exposure to Pb and As tends to increase the probability of intellectual disability when compared with exposure to a single metal ^155^. High Pb levels might affect mental and psychomotor development in children with high prenatal Cd exposure ^154, 156^. Co-exposure to high levels of Pb and Mn together in the prenatal period led to larger deficits in cognition and language development in children at two years of age compared with single-metal exposure ^157^. Pb exposure was associated with lower IQ scores but only among children with high blood Mn levels ^158^. In contrast to the synergetic effect between Pb and As, Pb along with Cd, Mn, or Hg exposure tends to work in an antagonistic way, given that the combined effect on cognitive deficits caused by MeHg and Pb exposure was less than additive ^159^.

In rodents, it has been reported that co-administration of two metals might increase the retention and redistribution of individual metals ^160^. Co-exposure of Se and Hg increased the retention of both metals and resulted in a redistribution of Hg in the blood and organs ^160^. Chandra *et al.* reported that oral intake of Mn and intraperitoneal administration of Pb in rats resulted in alternations in motor activity, learning ability, and biogenic amine levels and Pb levels in the brain, which were more severe than in rats exposed to either Mn or Pb alone ^161^. Similarly, rat brain weight decreased to a larger degree under conditions of co-exposure to both Mn and Pb than with either metal alone ^161^. An interaction of As/Pb has been reported to affect the central monaminergic systems in mice. The As/Pb mixture enhanced Pb accumulation but reduced As accumulation in the brain, decreased norepinephrine levels in the hippocampus, and increased serotonin levels in the midbrain and frontal cortex, when compared with single-metal exposure ^162^. In rats with oral consumption of mining waste (containing As, Cd, Mn, and Pb), accumulation of As and Mn was found in the brain, and dopamine release decreased with a long-standing polarization, when compared with the control ^163^. Mn may exacerbate the well-documented neurotoxic effects of Pb among children, particularly at younger ages ^154^. The metal mixture (As, Mn, and Pb) significantly decreased rat motor parameters compared with the single-metal exposure ^164^. Given that humans are exposed to multiple metals simultaneously in real life and neurotoxicity is commonly accompanied with metal overexposure, more studies are needed to investigate the health impacts of metal mixture in the near future to better understand their synergetic or antagonistic effect.

## Treatment for metal-induced neurotoxicity

Upon occurrence of metal poisoning, patients should be transferred out of the exposing environment first, followed by gastrointestinal decontamination. For example, a rapid decrease in blood plasma Mn levels has been noticed with discontinued Mn supplementation in children with cholestasis receiving total parental nutrition ^165^. However, this therapy might not work well for patients with chronic metal exposure, given that metals have already accumulated in the nervous system, bones, and other tissues rather than in the plasma. Currently, chelation therapy is a common treatment to remove additional metals in the body for both chronic and acute metal poisoning. Chelators include British antilewisite (BAL), succimer (DMSA), Prussian blue, calcium disodium edetate (CaNa _2_EDTA), and D-Penicillamine (Cuprimine) ^166^. However, the chelators themselves may have adverse effects, such as headache, fatigue, renal failure, nasal congestion, gastrointestinal side effects, and life-threatening hypocalcemia, to name a few ^166^. The specificity of these chelators and the dosage to use are the major concerns when performing chelation therapy. The discovery of specific metal exporters provides novel therapeutics for metal-induced neurotoxicity. For example, SLC30A10 is a newly identified Mn transporter facilitating efflux of excessive intracellular Mn; patients with mutations in the gene presented with dystonia, hypermanganesemia, parkinsonism, and manganism ^56, 77, 78, 167^. Although a study of its complete substrates has not been finished, this protein seems to transport Mn exclusively ^167, 168^. Pharmaceutical compounds enhancing the exporting activity of SLC30A10 will promote specifically Mn efflux but leave other metals untouched. This can bypass the side effects caused by chelation to a great degree. Unfortunately, specific transporters for different metals like SLC30A10 for Mn remain largely unknown. Future studies to identify metal-specific transporters are in great need to design therapeutics to treat metal-induced toxicity.

## Conclusions and future directions

Metals play an important role in our daily life as they are widely involved in numerous enzymatic activities. Some of them are essential at trace amounts; however, excessive amounts in the human body usually result in neurotoxicity. AD, ALS, autism, and PD are commonly associated with metal overexposure. Once metals have accumulated in the nervous system, oxidative stress, mitochondrial dysfunction, and protein misfolding are the most common deficits associated with metal-induced toxicity ^6– 13^. Once injured, neurons have to expend greater energy to synthesize neurotransmitters and maintain homeostasis. The increased burden combined with the neurotoxicity may lead to neuronal death. When some neurons are lost, the job has to be passed on to other neurons, initiating a vicious cycle of toxicity. Given that the nervous system does not regenerate as well as other systems do, the neurodegeneration and impairments usually become progressive with age, as typically seen in AD and PD. With the increase of lifespan among the general population, there is arguably a longer duration of exposure to greater levels of metals for individuals and potential increase in frequency of occurrence of neurological diseases. Accordingly, there is a growing demand to investigate the neurotoxicity resulting from metal exposure. Future studies need to focus more on the joint effect of metal mixture exposure, identifying specific transporters of each metal as well as developing target-specific therapeutics for patients with metal poisoning.
